# A bibliometric analysis of publications on obsessive–compulsive disorder using VOSviewer

**DOI:** 10.3389/fpsyt.2023.1136931

**Published:** 2023-05-18

**Authors:** Yimiao Tang, Xuewen Lu, Xin Wan, Maorong Hu

**Affiliations:** Department of Psychosomatic Medicine, The First Affiliated Hospital of Nanchang University, Nanchang, China

**Keywords:** bibliometric analysis, obsessive-compulsive disorder (OCD), visualization, VOSviewer, trends

## Abstract

**Background:**

Obsessive–compulsive disorder (OCD) is one of the top ten disabling diseases seriously affecting the health of population. Recently, studies on this disease significantly increased. However, only a few bibliometric analyses concerning this area have been reported. In this study, we used bibliometrics and visualization tools to examine the current state, hot topics and future trends in OCD research.

**Methods:**

Scientific publications regarding OCD were retrieved from the Web of Science Core Collection (WoSCC) database. The features of OCD research were further analyzed using VOSviewer.

**Results:**

A total of 24,552 publications and 65,296 authors in the field of OCD were retrieved from 2000 to 2022, showing an overall upward trend in publications over the past 22 years. One hundred and thirteen countries around the world had participated in the research. Among these countries, the developed countries such as the United States, England, and Canada were the crucial productive nations in this subject. As for institutions, the Harvard University, the University of London, and the University of California system were the leading institutions. Authors including Storch EA, Mataix-Cols D, and Stein DJ were the prolific authors. 1,949 journals are contributing to the OCD field, of which the top three are Biological Psychiatry (831 articles), European Neuropsychopharmacology (776 articles) and Psychiatric Research (648 articles). Research hotspots of OCD included pathogenesis, epidemiology, comorbidities, clinical features, and evaluation methods. COVID-19, mental health, functional connectivity, and genome-wide association were emerging trends in the field of OCD.

**Conclusion:**

This study integrates the bibliometric information on the current research status and emerging trends in OCD from a macro perspective. The findings can provide valuable insights into further research on OCD.

## Introduction

Obsessive–compulsive disorder (OCD) is one of the top ten mental diseases, with a high disability rate. Approximately 2% of the world’s population suffers from OCD and faces high medical costs (1). The clinical symptoms of OCD include constant obsession and repeated compulsion. Most OCD patients have coexisting mental diseases, such as depression, impulse control disorder, and most commonly, anxiety disorder (2). Many methods have been developed for OCD patients, including psychotherapy (cognitive behavioral techniques, etc.) (3), drug therapy (selective serotonin reuptake inhibitors, etc.) (4), and neuromodulation (repetitive transcranial magnetic stimulation, etc.) (5). Most patients benefit with standardized, evidence-based treatment. However, a significant proportion of them do not respond adequately, or have residual symptoms. Therefore, the etio-pathological mechanisms underlying OCD, and various therapeutic methods deserve further investigation.

Bibliometric analysis is an objective and efficient statistical method to describe published research studies within a certain field. Various scientific cartography programs can be used in bibliometric analysis. Furthermore, bibliometric analysis provides important quantitative information on how field-specific journals are distributed apart from the strength of collaborative linkages between the authors. Subsequently, researchers are provided the knowledge structure and trends in certain disciplines, further providing insights in future research directions (6, 7).

At present, although research concerning OCD is significantly increasing, bibliometric studies in the field of OCD are scarce. For instance, Parmar et al. (8) and Grover et al. (9) analyzed the number of publications and citations, but tended to ignore the cooperative link strength between the authors and institutions, proper visualizations of these associations were also lacking. In this study, we performed a new bibliometric analysis and visual analysis on OCD to provide a more intuitive illustration of the current and future trends in OCD research, thus providing new insights into future OCD research.

### Related works

Bibliometric analysis, which originated in the 1950s (10), has become increasingly popular in recent years because of the development of scientific literature databases (e.g., Scopus and Web of Science). With the easy obtainment of a large number of articles, publications on bibliometric analysis have also dramatically increased (11). As a relatively new statistical method, bibliometric analysis has been applied in the study of many fields, such as medicine (12–14), economics (15, 16), literature (17), engineering (18, 19) and others. By analyzing vast publications concerning a specific field, bibliometric analysis can reveal the associated number of publications and citations, keywords, themes, patterns of collaboration, emerging trends. This method also allows researchers to rapidly understand the dynamics of knowledge pertaining to a research area and derive new research ideas that may contribute to future research. In this study, the visualization software VOSviewer was used to more visually reveal the knowledge dynamics of the OCD field and predict future research directions via co-keyword analysis.

## Methods

On October 23, 2022, we retrieved and downloaded data from the Web of Science Core Collection (WoSCC) database. In the WoSCC database, we searched articles about obsessive–compulsive disorder between 1 January 2000 and 30 September 2022 used for this bibliometric analysis. The specific retrieval strategy is as follows: TS = [(obsessive–compulsive disorder) OR (obsessive compulsive disorder) OR OCD]; DOP = (2000-01-01/2022-09-30); language type = English; research areas = psychiatry OR behavioral sciences OR psychology OR neurosciences neurology. Import the basic information of downloaded literature into VOSviewer in TXT format for further OCD visual analysis, including author, country, journal, institution, keywords, etc.

## Results

### Analysis of annual publications

**Figure 1:**
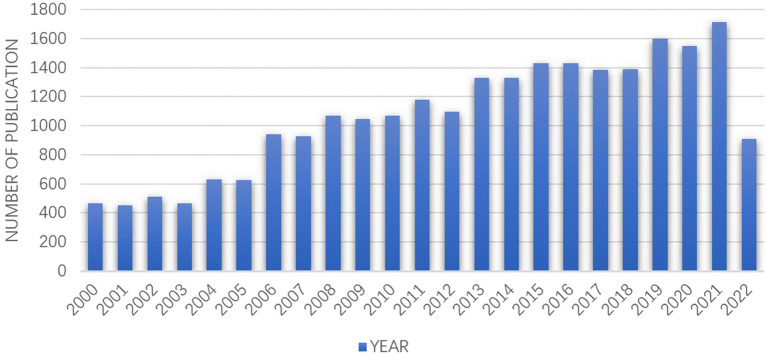
Trends in the number of OCD publications from 2000 to 2022.

From January 1, 2000, to September 30, 2022, a total of 24,552 OCD articles were retrieved in the WOSCC database, including 19,721 original research articles and 4,402 review articles. From Figure 1 we can see that the number of articles published in 2021 is the largest (1,713), and the overall trend is increasing year by year.

### Country/region, institution, author, journal citation analysis

**Figure 2:**
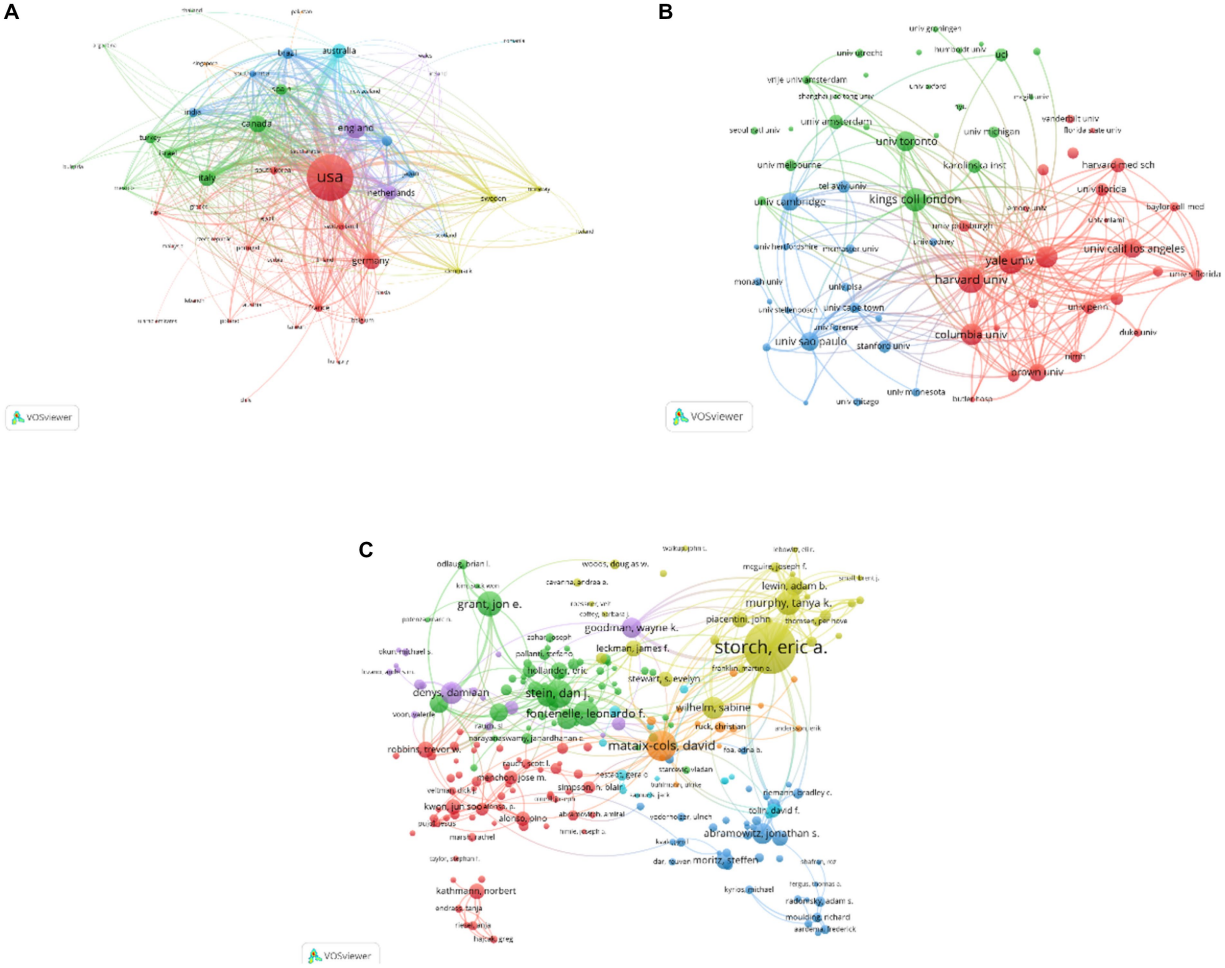
**(A)** Country/regional contributions in citations. **(B)** Contribution of the institution cited. **(C)** The author’s contribution in citations.

According to WoSCC, 113 countries had participated in research and published articles on OCD in the past 22 years. Table 1 listed the top 10 prolific countries/regions. The United States published the largest number of publications (9,740 article), followed by England (2,493 article), Canada (1868 articles), Germany (1812 articles), Italy (1725 articles), Australia (1,450 articles), Netherlands (1,450 articles), Spain (977 articles), Brazil (958 articles) and Turkey (881 articles). The results indicated that the developed countries were the most productive nations. Furthermore, Figure 2A represented that the United States, Canada, Italy, Germany, England and the Netherlands were at the center of the global cooperation network. It indicated that the United States had absolute influence in the field of OCD.

**Table 1:** List of the top 10 countries/regions with the highest studies related to OCD from 2000 to 2022.

Ranking	Country	Number of documents	%	Citation count
1st	United States	9,740	39.671	452,662
2nd	England	2,493	10.154	116,786
3rd	Canada	1868	7.608	70,621
4th	Germany	1812	7.380	76,660
5th	Italy	1725	7.026	53,303
6th	Australia	1,450	5.906	47,755
7th	Netherlands	1,346	5.482	53,828
8th	Spain	977	3.980	29,010
9th	Brazil	958	3.902	24,138
10th	Turkey	881	3.613	11,270

In addition, the research institutions were then analyzed. The top 10 productive institutions were shown in Table 2. Each organization published at least 631 articles related to OCD and more than 30% of all documents were produced by these top 10 institutions. Seven of institutions were from the United States, with the others coming from England and Canada. Harvard University ranked the first among these institutions, with 1,320 articles completed, followed by the University of London (1,165 articles) and the University of California System (1,022 articles). Moreover, the key research institutions and their link strength were assessed using institution network map. Harvard University, Yale University, Columbia University and King’s College London were all prominently displayed in Figure 2B. Among these institutions, Harvard University was dominant in the field of OCD.

**Table 2:** List of the top 10 institutions with the highest studies related to OCD from 2000 to 2022.

Ranking	Institution	Country	*n*	%
1st	Harvard University	United States	1,320	5.359
2nd	University of London	England	1,165	4.730
3rd	University of California System	United States	1,022	4.149
4th	State University System of Florida	United States	797	3.236
5th	Massachusetts General Hospital	United States	796	3.232
6h	Harvard Medical School	United States	734	2.980
7th	King’s College London	England	715	2.903
8th	Yale University	United States	691	2.805
9th	University of Toronto	Canada	645	2.619
10th	Columbia University	United States	631	2.562

Over the past two decades, lots of authors participated in the study of OCD. The top 10 authors in terms of documents, citations and H-index were displayed in Table 3. Storch EA was the most productive author (363 articles) and also was the most frequently cited author (9,640 citations), followed by Mataix-Cols D and Stein DJ. Besides, the H-index values of Mataix-Cols D and Stein DJ were both 66, higher than the H-index value of Storch EA. A map of co-authors and co-cited authors was also generated using VOSviewer. Based on the network of partnerships, we found that the top 3 authors with high centrality were also Storch EA, Mataix-Cols D and Stein DJ (Figure 2C).

**Table 3:** List of the top ten authors of OCD publications.

Authors	Number of publications	Count of citations	H-index
Storch EA	363	9,640	54
Mataix-Cols D	201	8,467	66
Stein DJ	182	6,200	66
Fontenelle LF	164	2,564	33
Grant JE	164	5,492	50
Murphy TK	160	6,088	48
Wilhelm S	142	4,461	46
Denys D	141	4,962	49
Miguel EC	133	2,593	45
Abramowitz JS	133	5,801	45

**Figure 3:**
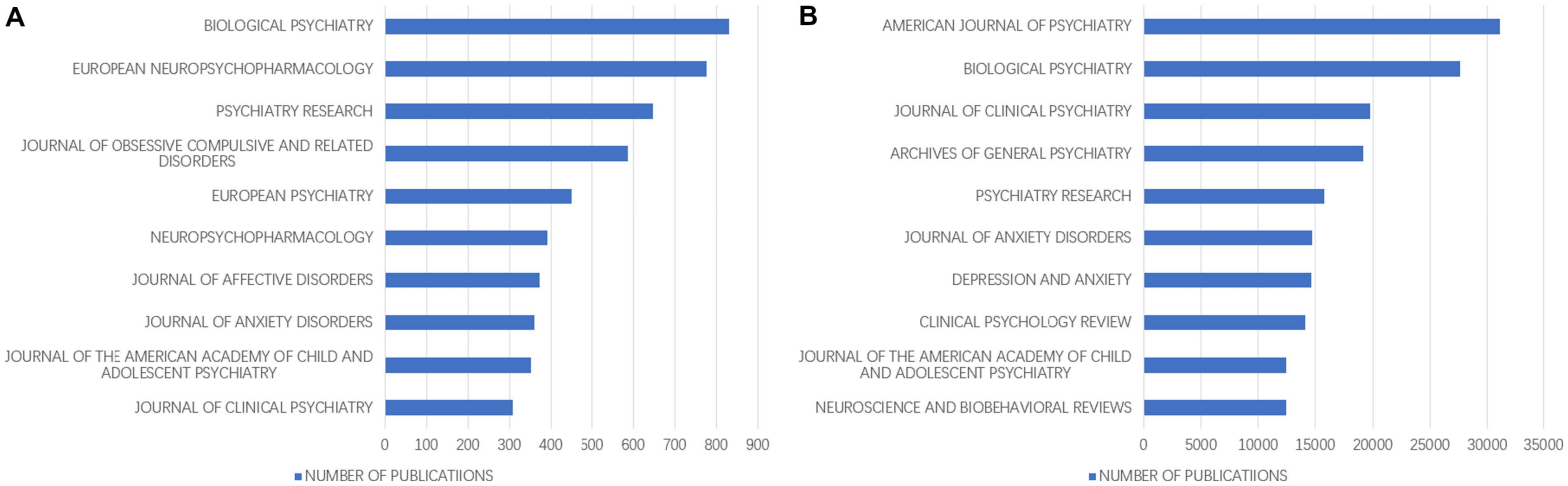
**(A)** Top 10 journals with the most published articles in the field of OCD. **(B)** The top 10 most cited journals in the field of OCD.

According to the journal analysis, a total of 1,949 journals published papers related to OCD from 2000 to 2022. And 229 of these journals published more than 20 articles on OCD. The journal of Biological Psychiatry had published the most articles in the field of OCD (831 articles) and had a high cited frequency (27,592 citations) (Figures 3A,B). Although the number of articles published by American Journal of Psychiatry was limited compared to the journal of Biological Psychiatry, the total citations and link strength ranked first among all journals (Figure 3B).

### Keywords analysis

**Figure 4:**
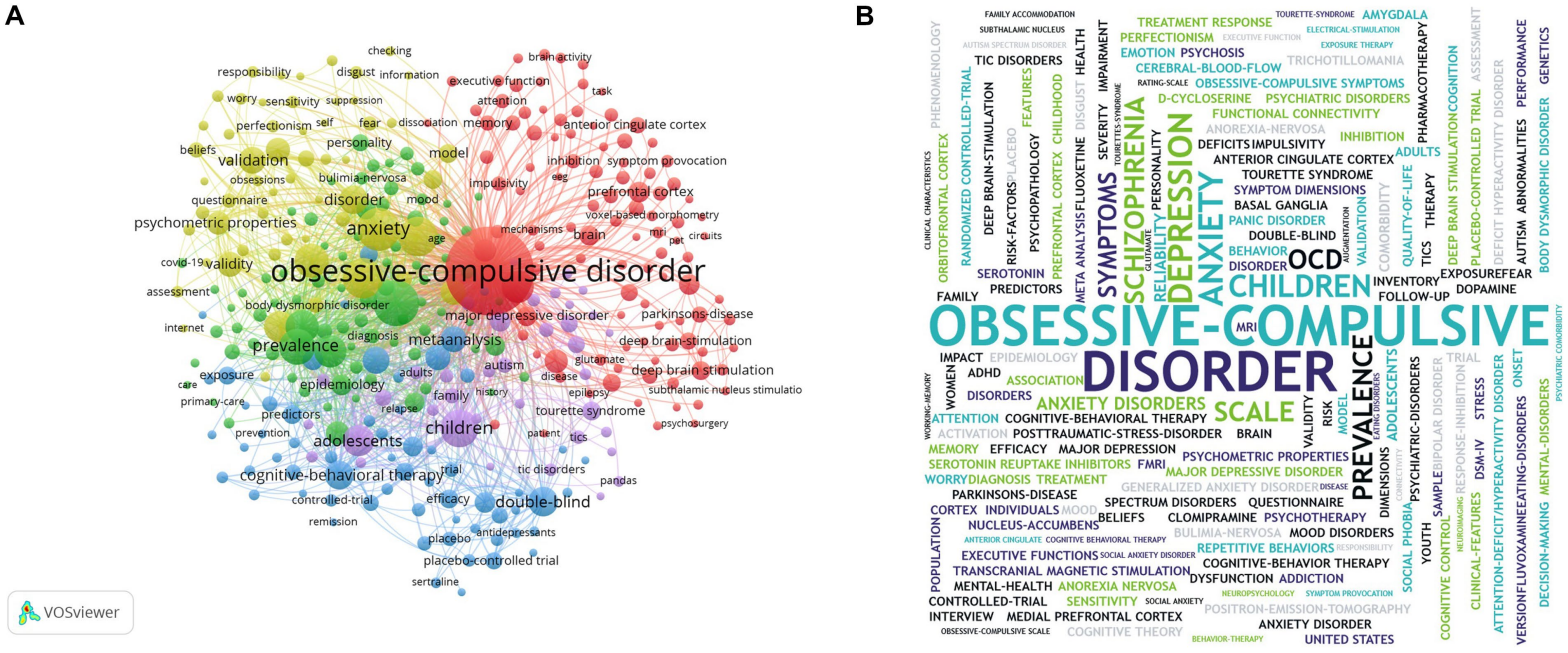
**(A)** The keyword co-occurrence map. **(B)** Word cloud. “obsessive–compulsive disorder,” “depression,” and “anxiety” occurred most commonly.

**Figure 5:**
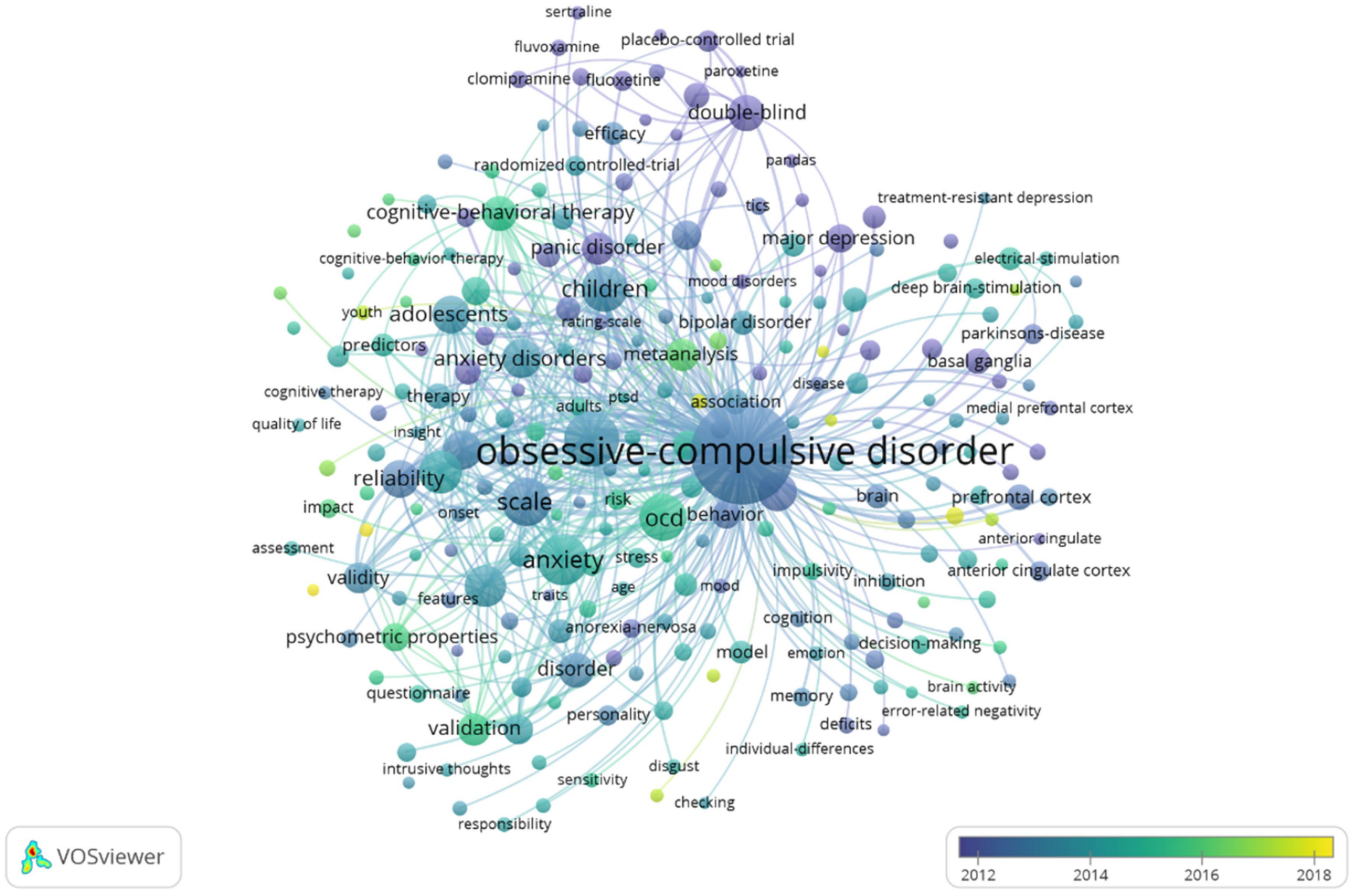
The overlay map.

Cluster analysis of keyword might be useful for identifying popular research topics and frontier scientific concerns. Therefore, the keyword co-occurrence function of VOSviewer was performed to cluster the hot spots on OCD into five, as shown in Figure 4A. Five colors were used to represent different clusters of research topics as follows: clinical features and diagnostic approaches of OCD (yellow), the mechanism of OCD (red), epidemiology and complications of OCD (green), treatment methods of OCD (blue), OCD in children and adolescents (purple). Meanwhile, a word cloud was created to display the frequency of keywords, the larger the node size, the higher the frequency (Figure 4B). In conclusion, the keyword of “obsessive–compulsive disorder” was the most frequently searched term, far superior to any other keywords. Subsequently, the appearance of keywords related to OCD over time were presented using Vosviewer’s overlayed visualization. A total number of 33,837 keywords in this field from 2000 to 2022 years were included in this study. As shown in Figure 5, there were 247 keywords, each receiving at least 140 searches, for displaying. Blue nodes represented the earlier appeared research keywords, while the yellow nodes represented the latest appeared research keywords. For example, the keyword of autism spectrum disaster, marked by yellow color, appeared in 2018. The other yellow keywords were COVID-19, mental health, functional connectivity, and the genome-wide association. It represented an emerging prospective of OCD researches in recent years.

## Discussion

Obsessive–compulsive disorder remains a serious public health problem, affecting the quality of life of patients. Patients with OCD often have suicidal ideation and tendency. Consequently, many various academic studies have been pursued in this field to determine probable mechanisms and appropriate treatment methods. In this research, we conducted a bibliometric analysis on OCD to summarize previous studies and provide insights into frontier directions. A total of 24,552 publications on OCD in the WOSCC database were included for analysis. The number of publications on OCD has increased dramatically in the past 22 years. Our finding is consistent with the research of Grover et al., who demonstrated that researchers increasingly paid attention to OCD.

Our analysis also indicates that researchers from 113 different countries had greatly contributed to the progression of the OCD field. The most prolific countries were the United States, England, Canada, Germany, Italy, Australia, and the Netherlands. Our finding is in line with previous bibliometric studies in OCD. In fact, the correlation between OCD incidence rates and local socioeconomic statuses could not be established (20). The high productivity of developed countries in terms of OCD research and publication may be ascribed to their higher research funding compared with that of developing countries (21). Furthermore, the United States was the leading country in the area of OCD. Most of the high-level research institutions were based in the United States. Other European countries, such as England and Germany, contributed greatly to the development of this discipline. However, the number of publications and citations were lower than the United States. Incidentally, the top ten countries did not include any Asian country, which suggests that those countries should increase their research funding and research quality and quantity.

Authorship analysis allowed us to determine the leading authors and influential research teams in the OCD field. Authors from developed countries dominated the list in terms of in the number of publications, citations, and H-index scores. Half of the top ten authors in the OCD field with the highest number of publications were from the United States. In addition, the research term of Storch EA, who worked in the Menninger Department of Psychiatry and Behavioral Sciences in the USA, was the leader in this field. In 2004, the Children’s Yale-Brown Obsessive–Compulsive Scale (CY-BOCS) was evaluated, further confirming the validity and reliability of the scale. This paper has far-reaching implications for the future diagnosis and efficacy evaluation of children with OCD (22). The development and progress of disciplines depend on communication and cooperation (23). Thus, in this study, we analyzed the strength of cooperative relationships between the research authors. The results showed that the strength and quantity of domestic cooperation were more prominent than those of international cooperation. Thus, international collaboration between authors should be further strengthened for disciplinary development.

The current hotspots of OCD research were identified using VOSviewer’s keyword co-occurrence function and then the results were broadly classified into five research directions: clinical features and assessment methods of OCD, mechanisms, epidemiology and comorbidity, treatment, and childhood and adolescent OCD. VOSviewer’s overlaid visualization was helpful in depicting the latest research directions. The yellow keywords on the map represent the emerging trends in OCD research. The relationship between coronavirus disease 2019 (COVID-19) and OCD and the research on genomics, the functional connectivity in OCD may be regarded as potential future directions according to the visualizations. COVID-19 has become a popular topic since 2019, especially since the mental health of many individuals has been affected (24). Nevertheless, research on the link between COVID-19 and OCD remains limited, and further research is essential. In addition, OCD is a highly heritable disease with abundant genetic changes. Identification of risk genes based on genome-wide association studies is helpful in understanding the mechanisms and treatments of OCD (25). In belief, further research on the functional connectivity and the genome-wide association will provide new perspectives into the pathogenesis of OCD.

Currently, increasing attention is being paid to mental health. OCD, depression (26), schizophrenia (27), premenstrual anxiety disorder (28), Asperger syndrome (29), and Alzheimer’s disease (30) are areas that are currently actively studied, and their number of publications is increasing yearly. Many psychiatric disorders have been analyzed using bibliometrics. Consistent with our study, the analyses demonstrate the dominance of developed countries in these areas. Developed Western countries, especially the United States, have a high number of publications. Furthermore, the authors with the highest number of publications are based on these developed Western countries. Collaborations among these highly productive countries are also higher compared with low-productivity countries. This finding may be explained by the high economic and academic status of developed countries, enabling better communication among them. Moreover, bibliometric analysis in each field described research hotspots and provide directions for the future development of related fields.

There were some restrictions in this study. Firstly, the analyzed data were obtained from only a single database, the WoSCC database. Publications listed in other databases were excluded from our study, which might have led to incomplete literature searches. Secondly, new articles from the next months of 2022 years might not be indexed due to the retrieval time. Thirdly, only English articles were included in this study. Finally, the studies of bibliometric analysis could only provide short-term predictions in the directions of the research field.

## Conclusion

In this study, we performed bibliometric analysis of OCD literature listed in the WoSCC database from 2000 to 2022 to reveal their research status, hotspots and emerging trends of OCD. The countries that dominated OCD research were developed Western countries, including the United States. Strengthened international cooperation may help further improve this field. Moreover, research on OCD in the past 22 years has mainly concentrated on pathogenesis, epidemiology, comorbidities, clinical features, evaluation methods, treatment, and OCD in children and adolescents. COVID-19, functional connectivity, and genome-wide association research in the field of OCD is limited and need further exploration in the future research, consequently contributing to the study and prevention of the pathogenesis of OCD.

## Data availability statement

The original contributions presented in the study are included in the article/supplementary material, further inquiries can be directed to the corresponding author/s.

## Author contributions

YT was responsible for the data collection, investigation, figures and tables construction and writing the original draft. MH, XL, and XW contributed to the discussion and final review and editing. All authors contributed to the article and approved the submitted version.

## Conflict of interest

The authors declare that the research was conducted in the absence of any commercial or financial relationships that could be construed as a potential conflict of interest.

## Publisher’s note

All claims expressed in this article are solely those of the authors and do not necessarily represent those of their affiliated organizations, or those of the publisher, the editors and the reviewers. Any product that may be evaluated in this article, or claim that may be made by its manufacturer, is not guaranteed or endorsed by the publisher.

## Abbreviations

OCD: Obsessive–compulsive disorder
WOSCC: Web of Science Core Collection
COVID-19: coronavirus disease 2019
